# Expression profile of long non-coding RNAs in pancreatic cancer and their clinical significance as biomarkers

**DOI:** 10.18632/oncotarget.5533

**Published:** 2015-10-02

**Authors:** Yingxue Wang, Zhihua Li, Shangyou Zheng, Yu Zhou, Lei Zhao, Huilin Ye, Xiaohui Zhao, Wenchao Gao, Zhiqiang Fu, Quanbo Zhou, Yimin Liu, Rufu Chen

**Affiliations:** ^1^ Guangdong Provincial Key Laboratory of Malignant Tumor Epigenetics and Gene Regulation, Department of Hepatopancreatobiliary Surgery, Sun Yat-sen Memorial Hospital, Sun Yat-sen University, Guangzhou, China 510120; ^2^ Department of Medical Oncology, Sun Yat-sen Memorial Hospital, Sun Yat-sen University, Guangzhou, China 510120; ^3^ Department of Radiation Oncology, Sun Yat-Sen University Cancer Center, Guangzhou, China 510060; ^4^ Department of Radiation Oncology, Sun Yat-sen Memorial Hospital, Sun Yat-sen University, Guangzhou, China 510120

**Keywords:** long non-coding RNA, pancreatic cancer, splice variant, microarray, biomarker

## Abstract

Long non-coding RNAs (lncRNAs) have shown great potential as powerful and non-invasive tumor markers. However, little is known about their value as biomarkers in pancreatic cancer (PC). We applied an Arraystar Human LncRNA Microarray which targeting 7419 lncRNAs to determine the lncRNA expression profile in PC and to screen the potential biomarkers. The most increased lncRNAs in PC tissues were HOTTIP-005, XLOC_006390, and RP11-567G11.1. Increased HOTTIP-005 and RP11-567G11.1 expression were poor prognostic factors for patients with PC (*n* = 144, *p* < 0.0001). The expression patterns of HOTTIP splice variants in PC were also detected. HOTTIP-005 and HOTTIP-001 were the first and second most increased HOTTIP splice variants, respectively. Plasma HDRF and RDRF (HOTTIP-005 and RP11-567G11.1 derived RNA fragments in plasma/serum) were present in stable form. Their levels were significantly increased in the patients with PC as compared to the healthy controls (*n* = 127 and 122 respectively, *p* < 0.0001) and the high levels were derived from PC. HDRF and RDRF levels are promising indicators for distinguishing patients with PC from those without PC. This study identified HOTTIP-005 and RP11-567G11.1 and their plasma fragments with the potential to be used as prognostic and diagnostic biomarkers of PC. Further large-scale prospective studies are needed to confirm our findings.

## INTRODUCTION

Pancreatic cancer (PC) is the fourth leading cause of cancer-related death globally [1]. Many patients are diagnosed at the advanced stages, where there is extensive local tumor invasion and early systemic dissemination, missing the best opportunity for curative surgery. Therefore, it is a highly lethal disease with very poor prognosis and a low 5-year survival rate (<6%) [2]. With emphasis on the dismal prognosis and survival, principally because effective biomarkers and therapeutic targets are still not identified. Despite the great efforts on the research of molecular alterations involved in PC [3–6], due to the sophistication and heterogeneity of this disease [7], the situation for PC is still deplorable. Thus, in-depth research of the genetic alterations and underlying molecular mechanism of PC is still an urgent issue.

Protein-coding genes in the human genome have been well-studied in the last few decades. However, while at least 90% of the human genome is actively transcribed [8], the protein-coding genes account for only 1.5% of the genome [9]. There has been an explosion of research into the possible functional roles of the remaining 98% of the human genome that does not encode proteins, namely the non-coding RNAs (ncRNAs). These so-called ncRNAs are arbitrarily separated into long ncRNAs (lncRNAs) and short ncRNAs including microRNAs (miRNAs) on the basis of their size [10]. LncRNAs are commonly described as RNA molecules > 200 nucleotides in length. Recent studies have postulated that lncRNAs are of crucial functional importance for cell fate determination and disease occurrence [11, 12]. Growing evidence is supporting the involvement of lncRNAs in tumorigenesis and tumor progression [13–16]. They may modulate cancer initiation and progression by affecting several biological pathways [17, 18]. As they are implicated as tumor suppressors and oncogenes, some lncRNAs are promising biomarkers and diagnostic and treatment targets. For example, metastasis-associated lung adenocarcinoma transcript 1 (MALAT-1) appears to be a potential diagnostic and prognostic marker of non–small cell lung cancer [13, 19]. Another study described MALAT-1–derived fragments as a novel plasma-based biomarker for diagnosing prostate cancer [20]. Differential display code 3 (DD3/PCA3), a prostate-specific lncRNA, acted as a biomarker that could be detected in the urine of patients with prostate cancer [21, 22]. However, only a few studies have investigated the relationship between lncRNAs and PC [23–26]. The expression and function of most lncRNAs in PC and their clinical significance remain unknown.

In the present study, we explored the lncRNA expression profile in PC using Arraystar Human LncRNA Microarray which targeting 7419 lncRNA and validated our results in cancer tissues and their paired adjacent non-tumorous tissues. Next, we analyzed the relationship between the aberrantly expressed lncRNAs and the clinicopathological factors of patients with PC. To find effective biomarkers, we detected the expression levels of two lncRNA fragments in blood from the patients with PC. Our results provide candidate diagnostic and prognostic biomarkers for PC.

## RESULTS

### LncRNA expression profile in PC tissues relative to non-tumorous tissues

**Figure 1:**
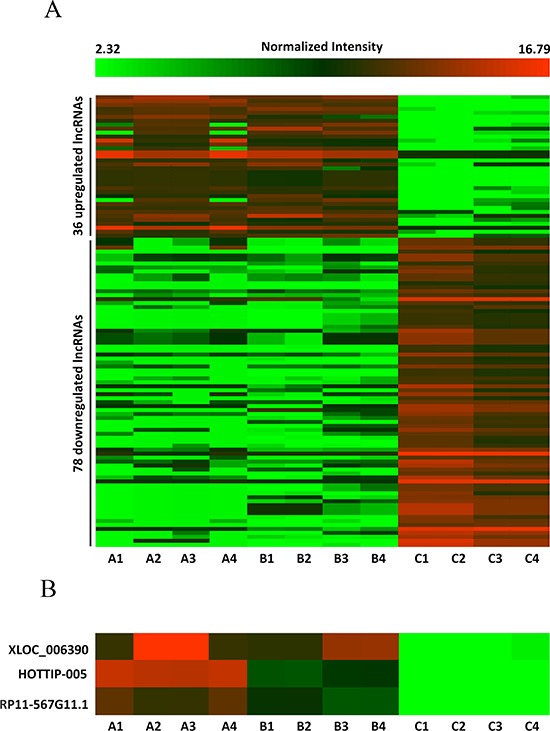
Differences in lncRNA expression profiles between PC and chronic pancreatitis tissues **A.** Heat map representing hierarchical clustering of distinct lncRNA expression profiling in PC tissues and chronic pancreatitis tissues (fold change > 2.5). Red color scale, higher expression; green color scale, lower expression. **B.** High-resolution heat map showing that XLOC_006390, HOTTIP-005, and RP11-567G11.1 were the most upregulated lncRNAs in the PC tissues. A1–4: Diabetes mellitus–associated PC; B1–4: PC without diabetes mellitus; C1–4: chronic pancreatitis.

We conducted lncRNA microarray analysis which was mentioned previous [27] on eight PC tissues and four chronic pancreatitis tissues utilizing a microarray targeting 7419 lncRNAs (Arraystar Human LncRNA Microarray V3; Agilent Technology, Santa Clara, CA). As pancreatitis is a known risk factor for pancreatic cancer [28, 29], we chose chronic pancreatitis (CP) tissues as a control to avoid the interference of inflammation. Analysis of the normalized lncRNA expression levels by Student's t test revealed significantly different lncRNA expression patterns between PC tissues and chronic pancreatitis tissues (Figure 1A). To identify the lncRNAs that were potential biomarkers, we further extracted 33 lncRNAs with fold change > 3 (Table 1); seven and 26 were upregulated and downregulated, respectively, in the tumor tissues. The upregulated lncRNAs with the greatest fold change, in descending order, were XLOC_006390, HOTTIP-005, and RP11-567G11.1 and were selected for validation. (Table 1, Figure 1B).

**Table 1:** LncRNAs which were > 3-fold differentially expressed in PC tissues as compared with chronic pancreatitis tissues

Gene Symbol	Chromosome	RNA length	Regulation	Fold change	*P* value
XLOC_006390	chr7	4899	up	4.24	5.29E-07
HOTTIP-005	chr7	2285	up	4.13	1.65E-05
RP11-567G11.1	chr3	246	up	3.78	1.47E-07
XLOC_012784	chr18	2402	up	3.71	9.24E-07
LINC00460	chr13	935	up	3.25	7.88E-11
CTD-2547H18.1	chr11	568	up	3.15	0.00905
RP3-395M20.9	chr1	626	up	3.00	1.08E-07
TMED11P	chr4	533	down	5.45	2.46E-09
TMED11P	chr4	204	down	4.34	7.17E-07
RP5-894D12.3	chr6	346	down	4.26	8.92E-09
HMlincRNA767	chr5	18267	down	4.25	6.12E-09
RP11-331F4.4	chr16	697	down	3.93	1.93E-10
RP4-809F18.1	chr12	1310	down	3.89	3.70E-15
LINC00426	chr13	276	down	3.83	1.00E-10
RP11-753D20.4	chr14	2352	down	3.71	4.15E-07
LOC389023	chr2	744	down	3.67	6.68E-07
AC011306.2	chr2	900	down	3.64	9.35E-10
RP11-404P21.3	chr14	738	down	3.48	6.57E-09
BC038546	chr8	907	down	3.42	0.0001
RP11-680B3.2	chr3	1414	down	3.36	6.49E-08
RP11-331F4.4	chr16	527	down	3.32	3.13E-13
COL6A4P1	chr3	1251	down	3.31	7.75E-07
RP11-76I14.1	chr2	1378	down	3.25	2.09E-07
AC114877.3	chr3	582	down	3.20	3.88E-08
RP5-894D12.3	chr6	318	down	3.17	7.44E-07
XLOC_002309	chr2	780	down	3.15	3.37E-06
XLOC_005355	chr6	585	down	3.07	5.15E-09
CELP	chr9	1216	down	3.05	5.93E-12
XLOC_005737	chr6	1374	down	3.04	1.05E-08
XLOC_011288	chr15	240	down	3.03	2.48E-07
AC007349.4	chr7	587	down	3.03	6.20E-06
chr12:53675775-53689275	chr12	13500	down	3.01	1.32E-06
LINC00316	chr21	455	down	3.01	2.02E-08

### HOTTIP-005 was validated as the most increased splice variant of HOTTIP in PC tissues

**Figure 2:**
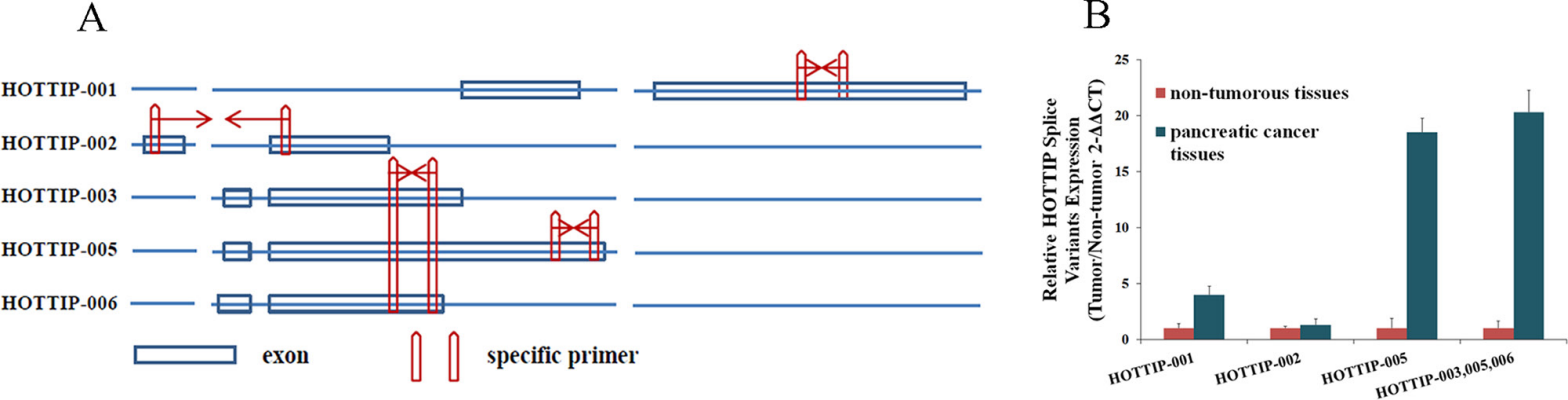
Identification of HOTTIP splice variant expression levels **A.** Five HOTTIP splice variants and the representative diagrams of their sequences; specific qRT-PCR primers were designed for HOTTIP-001, HOTTIP-002, and HOTTIP-005. The HOTTIP-003 and HOTTIP-006 sequences completely or closely matched that of HOTTIP-005, and we designed a primer that was simultaneously specific for HOTTIP-003, HOTTIP-005, and HOTTIP-006. **B.** Relative expression level of HOTTIP-001, HOTTIP-002, HOTTIP-003, HOTTIP-005, and HOTTIP-006 in PC tissues compared to adjacent non-tumorous tissues (*n* = 10). HOTTIP-005 was the most upregulated splice variant in PC tissues, followed by HOTTIP-001.β-actin was used as the normalization control.

To date, five splice variants of HOTTIP have been reported: HOTTIP-001, HOTTIP-002, HOTTIP-003, HOTTIP-005, and HOTTIP-006 (http://asia.ensembl.org) (Figure 2A). The lncRNA microarray analysis showed that HOTTIP-005 was significantly increased, as detected by the HOTTIP-005–specific probe. To validate the finding that HOTTIP-005 was the most increased HOTTIP slice variant in PC tissues, we designed specific quantitative real-time PCR (qRT-PCR) primers for HOTTIP-001, HOTTIP-002, and HOTTIP-005, respectively. As the HOTTIP-003 and HOTTIP-006 sequences completely or closely match that of HOTTIP-005, we designed a primer that was simultaneously specific to HOTTIP-003, HOTTIP-005, and HOTTIP-006, i.e., HOTTIP-003,005,006–specific (Figure 2A). We performed the qRT-PCR using each primer pair in PC tissues and the adjacent non-tumorous tissues (*n* = 10). β-actin was used as the normalization control. Relative expression analysis showed that HOTTIP-005 was the most increased splice variant in the PC tissues, followed by HOTTIP-001, while the total expression of HOTTIP-002, HOTTIP-003, and HOTTIP-006 accounted for a very small proportion of the increased splice variants (Figure 2B). We confirmed that the HOTTIP-001, HOTTIP-002, HOTTIP-005, and HOTTIP-003,005,006 amplicons were specific by sequencing the qRT-PCR products (Supplementary Figure S1).

### HOTTIP-005, XLOC_006390, and RP11-567G11.1 expression in normal and PC cell lines

**Figure 3:**
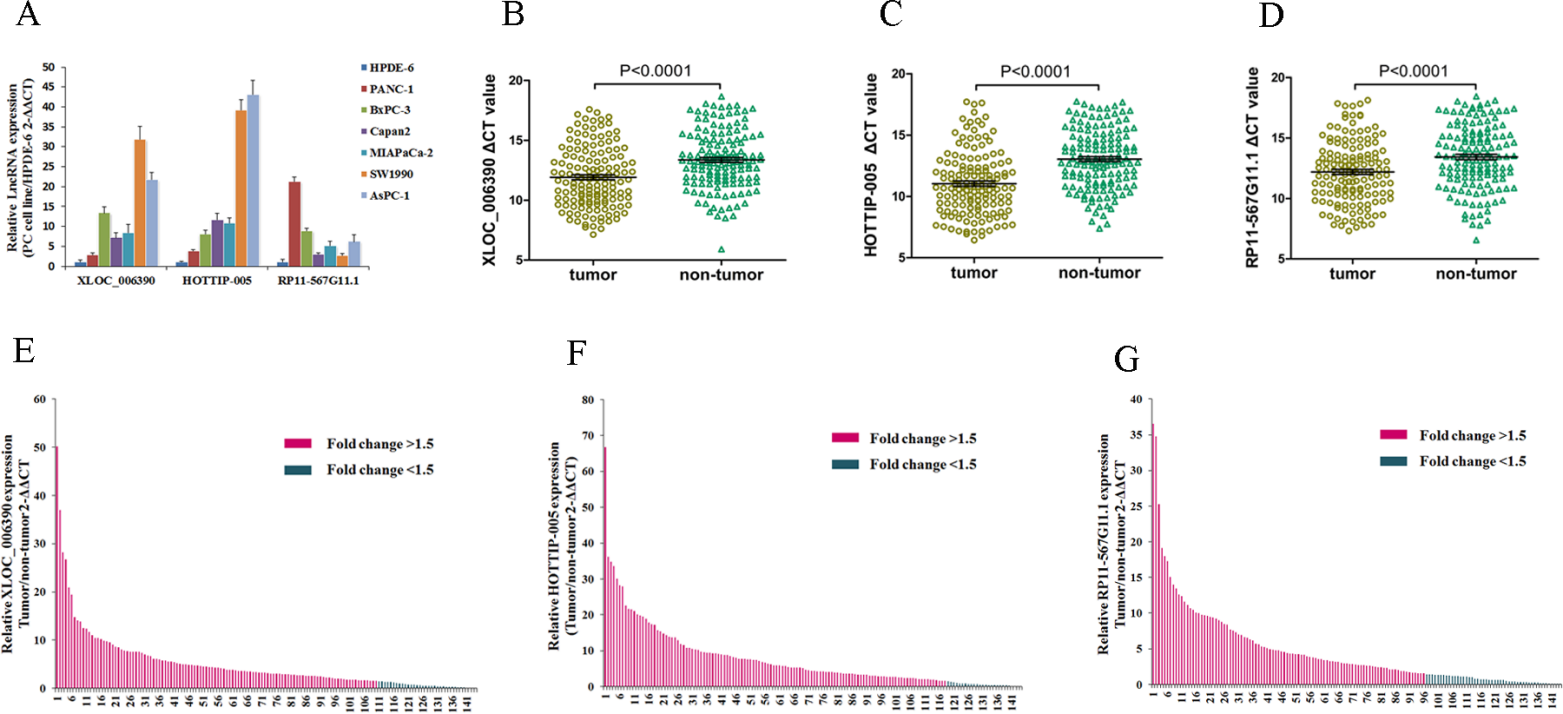
XLOC_006390, HOTTIP-005, and RP11-567G11.1 expression were increased in PC cell lines and tissues **A.** qRT-PCR evaluation of XLOC_006390, HOTTIP-005, and RP11-567G11.1 expression in PC cell lines as compared with the HPDE6 cell line. **B.** XLOC_006390, **C.** HOTTIP-005, and **D.** RP11-567G11.1 expression levels in PC tissues were all significantly higher than that in the non-tumorous tissues (*n* = 144, *P* < 0.0001). Smaller ΔCT value indicates higher expression. β-actin was used as the normalization control. **E.** XLOC_006390, **F.** HOTTIP-005, and **G.** RP11-567G11.1 relative gene expression was determined using the ΔCT method; data are presented as 2 − ΔΔCT. Fold change > 1.5 was deemed upregulation. XLOC_006390, HOTTIP-005, and RP11-567G11.1 were upregulated in 76.4% (110/144), 81.9% (118/144), and 66.7% (96/144) of PC tissue samples, respectively.

We examined HOTTIP-005, XLOC_006390, and RP11-567G11.1 expression in a normal cell line (HPDE6) and six PC cell lines (PANC-1, BxPC-3, Capan2, MIAPaCa-2, SW1990, AsPC-1) using qRT-PCR. Compared with the HPDE6 cells, all PC cell lines had higher HOTTIP-005, XLOC_006390, and RP11-567G11.1 expression; HOTTIP-005, XLOC_006390, and RP11-567G11.1 expression was highest in the AsPC-1, SW1990, and PANC-1 cells, respectively (Figure 3A).

### HOTTIP-005, XLOC_006390, and RP11-567G11.1 expression was increased in PC tissues

As an initial step, we sought to identify lncRNAs that were overexpressed in PC. As XLOC_006390, HOTTIP-005, and RP11-567G11.1 were the three most upregulated lncRNAs in PC tissues, we detected their expression levels in 144 cancer tissues and the paired adjacent non-tumorous tissues using qRT-PCR. The XLOC_006390, HOTTIP-005, and RP11-567G11.1 expression levels in the cancer tissues were all significantly higher than that in the non-tumorous tissues (Figure 3B–3D). The qRT-PCR sequencing of the products revealed that the HOTTIP-005, XLOC_006390, and RP11-567G11.1 sequences were consistent with that in the database (Supplementary Figure S2).

### Correlation between PC clinical characteristics and HOTTIP-005, XLOC_006390, and RP11-567G11.1 expression

Fold change > 1.5 was deemed high expression; we found that HOTTIP-005, XLOC_006390, and RP11-567G11.1 were increased in 81.9% (118/144), 76.4% (110/144), and 66.7% (96/144) of PC tissues, respectively (Figure 3E–3G). Next, we determined whether HOTTIP-005, XLOC_006390, and RP11-567G11.1 expression was associated with the clinicopathological features of PC. Table 2 shows that HOTTIP-005 overexpression was associated with degree of pathological differentiation, lymphatic metastasis, and early recurrence. No statistical correlation with age, sex, carbohydrate antigen (CA) 19-9 level, T stage, or neural invasion was observed. XLOC_006390 expression levels were associated with neural invasion, while RP11-567G11.1 expression was associated with lymphatic metastasis and T stage. The other clinical characteristics showed no statistical relationship with XLOC_006390 and RP11-567G11.1 expression.

**Table 2:** Correlation of lncRNA expression and clinicopathological factors of patients with PC

Characteristics		No. of patients	HOTTIP-005			XLOC_006390			RP11-567G11.1		
			low	high	*P* value	low	high	*P* value	low	high	*P* value
Total		144	26	118		34	110		48	96	
Age (years)	<60	70	12	58	0.782	16	54	0.836	23	47	0.906
	≥60	74	14	60		18	56		25	49	
Gender	Male	102	18	84	0.843	21	81	0.183	32	70	0.437
	Female	42	8	34		13	29		16	26	
CA19-9 (U/ml)	≤34	30	5	25	0.824	10	20	0.159	11	19	0.663
	>34	114	21	93		24	90		37	77	
Differentiation	Well	21	10	11	**0.001**	8	13	0.188	11	10	0.132
	Moderate	68	9	59		16	52		20	48	
	Poor	55	7	48		10	45		17	38	
T stage	T1	19	6	13	0.246	7	12	0.344	12	7	**0.012**
	T2	52	9	43		11	41		16	36	
	T3	73	11	62		16	57		20	53	
N stage	N0	59	16	43	**0.018**	15	44	0.670	26	33	**0.023**
	N1	85	10	75		19	66		22	63	
Neural invasion	Negative	69	14	55	0.504	23	46	**0.008**	20	49	0.288
	Positive	75	12	63		11	64		28	47	
Early recurrence	No	62	17	45	**0.011**	14	48	0.800	18	44	0.341
	Yes	82	9	73		20	62		30	52	

Bold values are statistically significant (*p* < 0.05).

### Association between prognosis and HOTTIP-005, XLOC_006390, and RP11-567G11.1 expression

Univariate analysis of overall survival revealed that T stage (*P* = 0.038), Lymph node metastasis (*P* = 0.011), Early recurrence (*P* = 0.009), HOTTIP-005 expression (*P* < 0.001) and RP11-567G11.1 expression (*P* < 0.001) were prognostic indicators (Table 3). Multivariate analysis indicated that Lymph node metastasis (*P* = 0.009), Early recurrence (*P* = 0.007), HOTTIP-005 expression (*P* < 0.001) and RP11-567G11.1 expression (*P* = 0.003) were independent prognostic indicators for overall survival of patients with PC (Table 3).

**Table 3:** Univariate and multivariate Cox regression of prognostic factors for overall survival in pancreatic cancer

Parameter	Univariate analysis			Multivariate analysis		
	HR	95% CI	*P*	HR	95% CI	*P*
Age (<60 *vs*. ≥60)	0.975	0.597–1.867	0.793			
Gender (Female *vs.* male)	1. 527	0.785–1.986	0.492			
CA19-9 (U/ml) (≤34 *vs*. > 34)	0.661	0.389–1.024	0.157			
Differentiation(Well *vs*. moderate *vs*. poor)	0.978	0.953–1.003	0.085			
T stage (T1 *vs*. T2 *vs*. T3)	1.606	1.184–2.192	**0.038**	1.389	1.236–3.546	0.072
N stage (N0 *vs*. N1)	1.698	1.130–2.552	**0.011**	1.992	1.262–3.142	**0.009**
Neural invasion(Negative *vs*. Positive)	2.451	1. 459–4. 098	0.063	1.613	0. 726–2. 527	0.162
Early recurrence (No *vs*. Yes)	2. 391	1.431–4.159	**0.009**	2.128	1.233–3.673	**0.007**
HOTTIP-005 expression (low vs. high)	3.881	2.202–6.840	**<0.001**	2.589	1.385–4.839	**<0.001**
XLOC_006390 expression (Low *vs*. high)	1.464	0.935–2.292	0.096	0.930	0.582–1.486	0.762
RP11-567G11.1 expression (Low *vs*. high)	3.279	2.148–5.004	**<0.001**	2.371	1.516–3.708	**0.003**

HR hazard ratio; 95% CI 95% confidence interval

Bold values are statistically significant (*P* < 0.05)

**Figure 4:**
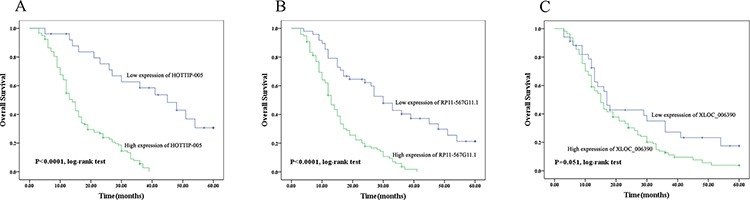
Prognostic significance of HOTTIP-005, XLOC_006390, and RP11-567G11.1 expression Overall survival was compared between the groups with high and low expression of **A.** HOTTIP-005 (*P* < 0.0001, *n* = 118 and 26), **B.** RP11-567G11.1 (*P* < 0.0001, *n* = 110 and 34), and **C.** XLOC_006390 (*P* = 0.051, *n* = 96 and 48). The median survival time of high and low expression subgroup of HOTTIP-005, RP11-567G11.1, and XLOC_006390 is 14 and 45 months, 13 and 30 months, and 15 and 17 months respectively. Kaplan–Meier survival estimates and log-rank testing determined that increased HOTTIP-005 and RP11-567G11.1 expression predicts poor prognosis in PC.

Kaplan–Meier survival analysis and log-rank testing showed that patients with low HOTTIP-005 expression had significantly increased overall survival as compared with patients with high HOTTIP-005 expression (*n* = 26 and 118, *P* < 0.0001). The same observation was made for RP11-567G11.1 expression (*n* = 34 and 110, *P* < 0.0001) (Figure 4A, 4B). While the overall survival rate was not associated with XLOC_006390 expression (*n* = 48 and 96, *P* = 0.051) (Figure 4C). The median survival time of high and low expression subgroup of HOTTIP-005, RP11-567G11.1, and XLOC_006390 is 14 and 45 months, 13 and 30 months, and 15 and 17 months respectively. These data indicate that increased HOTTIP-005 and RP11-567G11.1 expression are poor prognostic factors for patients with PC.

### Determining whether plasma/serum lncRNAs can serve as biomarkers

#### General characterization of plasma/serum lncRNA fragments

**Figure 5:**
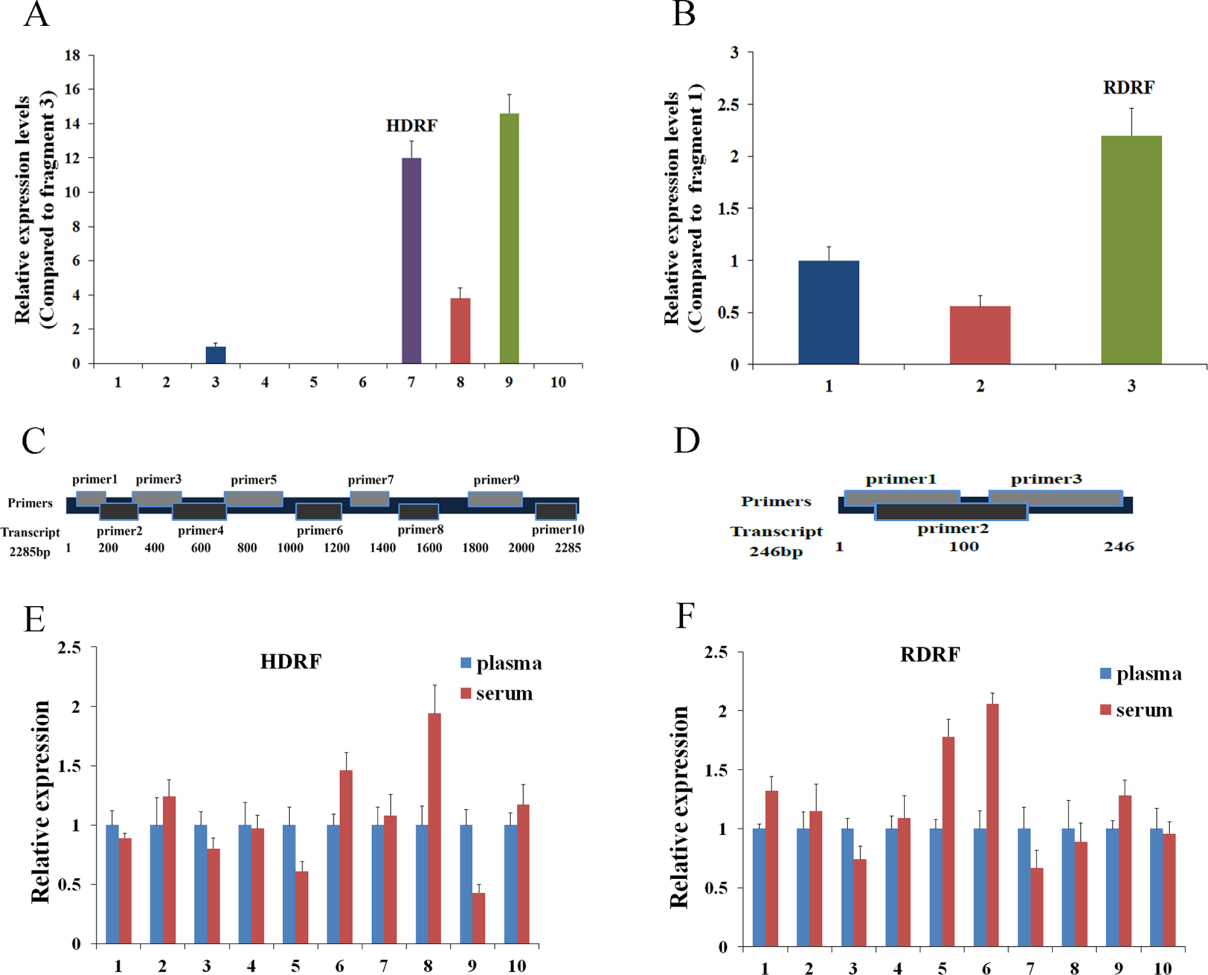
General characterization of plasma/serum lncRNA fragments **A.** qRT-PCR assessment of HOTTIP-005 and **B.** RP11-567G11.1 fragment expression in 10 PC plasma samples. Amplicon 9 of HOTTIP-005 and amplicon 3 of RP11-567G11.1 were the most highly expressed fragments. **C.** HOTTIP-005 and **D.** RP11-567G11.1 primer design schematics. Comparison of **E.** HDRF and **F.** RDRF levels in plasma and serum. We used qRT-PCR to assess their expression levels in 10 independent samples. GAPDH was used as the normalization control.

As the expression of HOTTIP-005 was the highest in PC tissues, and the RP11-567G11.1 sequence length was only 246 bp, rendering both lncRNAs easily detectable, we detected the HOTTIP-005 and RP11-567G11.1 expression levels in the plasma/serum from patients with PC to determine whether they could serve as effective and minimally invasive biomarkers. As there is no exact description of the general characterization of lncRNAs in plasma, we designed 10 and three primer pairs for HOTTIP-005 and RP11-567G11.1, respectively, to ensure that the amplicons would account for almost the entire transcript ( 1, Figure 5C, 5D); primer pairs 7 and 10 were HOTTIP-005–specific. Detection of the HOTTIP-005 and RP11-567G11.1 fragments in 10 PC plasma/serum samples revealed that the expression levels differed significantly among the fragments, but that the expression level of each fragment was relatively stable in each sample (Figure 5A, 5B).

Although the expression level of fragment 9 was the highest, we selected fragment 7, which was HOTTIP-005–specific, for further study. We also selected the RP11-567G11.1 fragment with the highest plasma expression level for further study. The fragments were designated HOTTIP-005–derived RNA fragment (HDRF) and RP11-567G11.1–derived RNA fragment (RDRF). In addition, sequencing the qRT-PCR products, which were termed HDRF and RDRF, proved that the amplicons were the intended fragments derived from HOTTIP-005 and RP11-567G11.1 (Supplementary Figure S3). Additionally, the targeted lncRNA fragments were stable in both plasma (EDTA as anticoagulant) and serum (Figure 5E, 5F). This result suggests that plasma and serum are both suitable for investigating lncRNA fragments as blood-based biomarkers.

**Figure 6:**
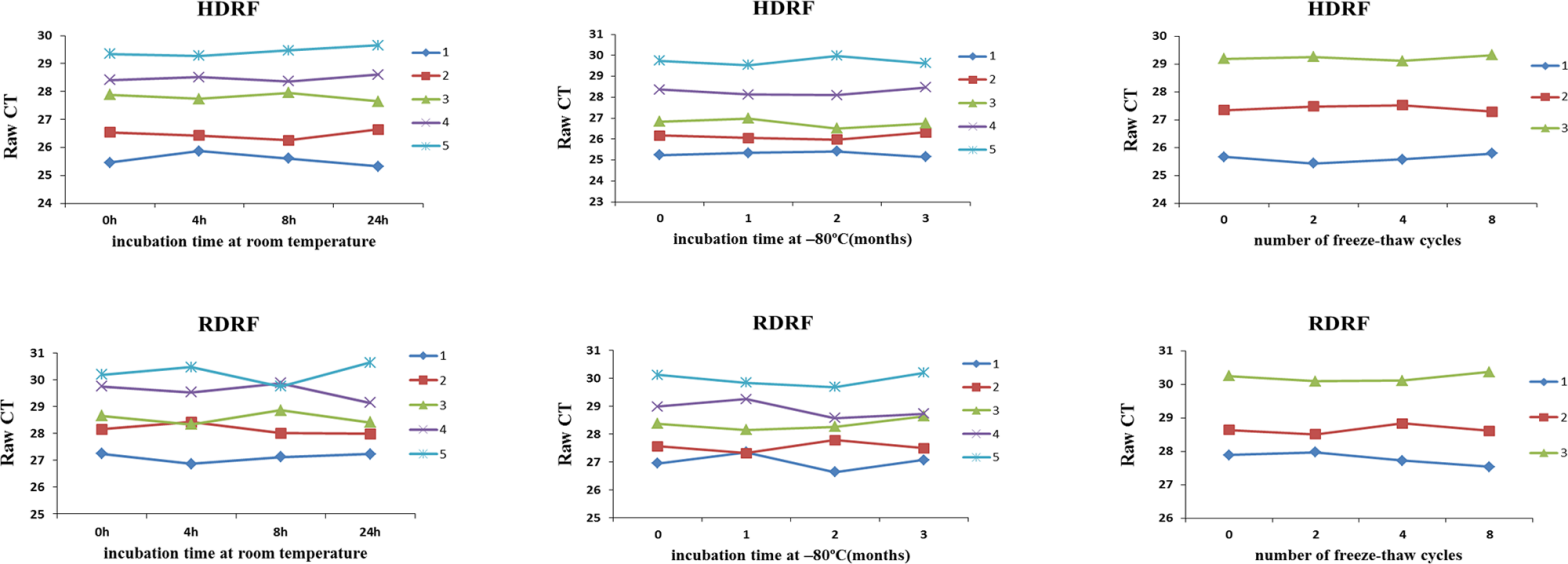
Stability of human plasma lncRNA fragments HDRF (top) and RDRF (bottom) plasma levels remained stable following prolonged exposure to room temperature or −80°C, and multiple freeze–thawing.

We next investigated the stability of plasma HDRF and RDRF. The plasma from 5 patients with PC was exposed to harsh conditions, including storage at room temperature for 4, 8, and 24 h, incubation at −80°C, and eight repetitive freeze–thaw cycles. Overall, the treatments did not apparently affect the HDRF and RDRF, which could be stably detected from all samples (Figure 6). Therefore, our study suggests that plasma lncRNA fragments are stable and detectable, which provides a foundation for their evaluation as useful and minimally invasive cancer biomarkers.

#### Validation of the source of plasma HDRF and RDRF

**Figure 7:**
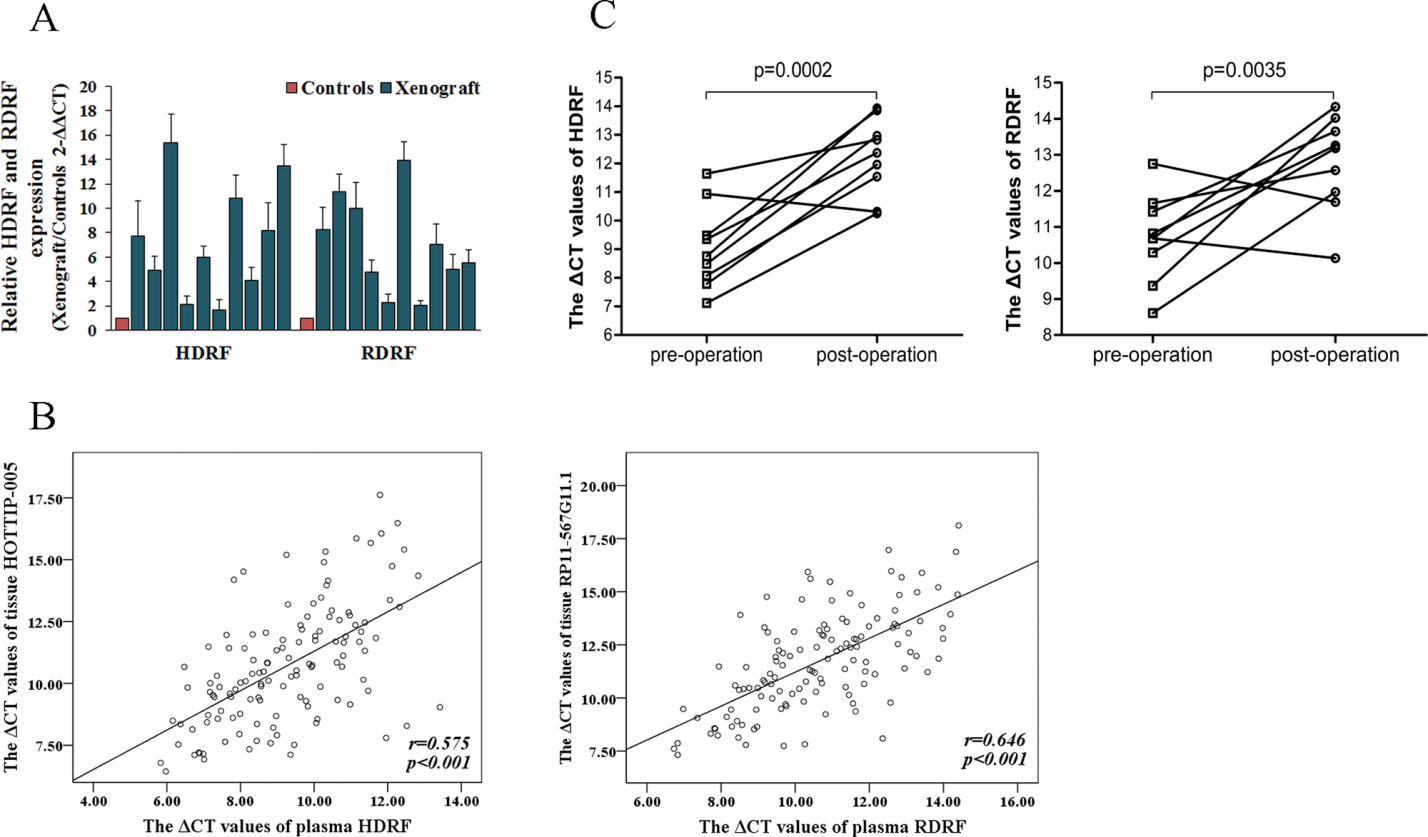
Source of human plasma lncRNA fragments **A.** HDRF and RDRF detected in xenograft plasma (*n* = 10), all at distinctly higher levels than that of the controls (*n* = 10). **B.** Spearman's rank correlation scatter plot of HOTTIP-005 and RP11-567G11.1 levels in PC tissues and plasma. Data were presented as ΔCt values. Expression levels of HOTTIP-005 and RP11-567G11.1 in PC tissues were significantly correlated with HDRF and RDRF levels in plasma. **C.** HDRF and RDRF expression levels in patients with PC before surgery and seven days after surgery (*n* = 9). HDRF and RDRF levels were all significantly decreased after surgery.

First, we performed xenograft experiments to compare the plasma HDRF and RDRF expression levels of PC xenografts with control mice, where blood was collected at four weeks after injection. The qRT-PCR results demonstrated that the plasma HDRF and RDRF expression levels were significantly increased when PC developed (Figure 7A).

Second, we analyzed the correlation between HOTTIP-005 and RP11-567G11.1 expression level in PC tissues and HDRF and RDRF expression level in plasma. As shown in Figure 7B, a significant correlation was observed for HOTTIP-005 and HDRF (*r* = 0.575, *P* < 0.001), RP11-567G11.1 and RDRF (*r* = 0.646, *P* < 0.001), respectively.

Third, to further validate the high expression levels of the PC-derived HDRF and RDRF, we assessed the HDRF and RDRF expression levels in patients with PC before surgery and seven days after surgery (*n* = 9), and found that the plasma HDRF and RDRF levels were significantly decreased after surgery (Figure 7C). These findings all suggest that the high levels of HDRF and RDRF expression are derived from PC.

#### HDRF and RDRF as plasma-based biomarkers for diagnosing PC

**Figure 8:**
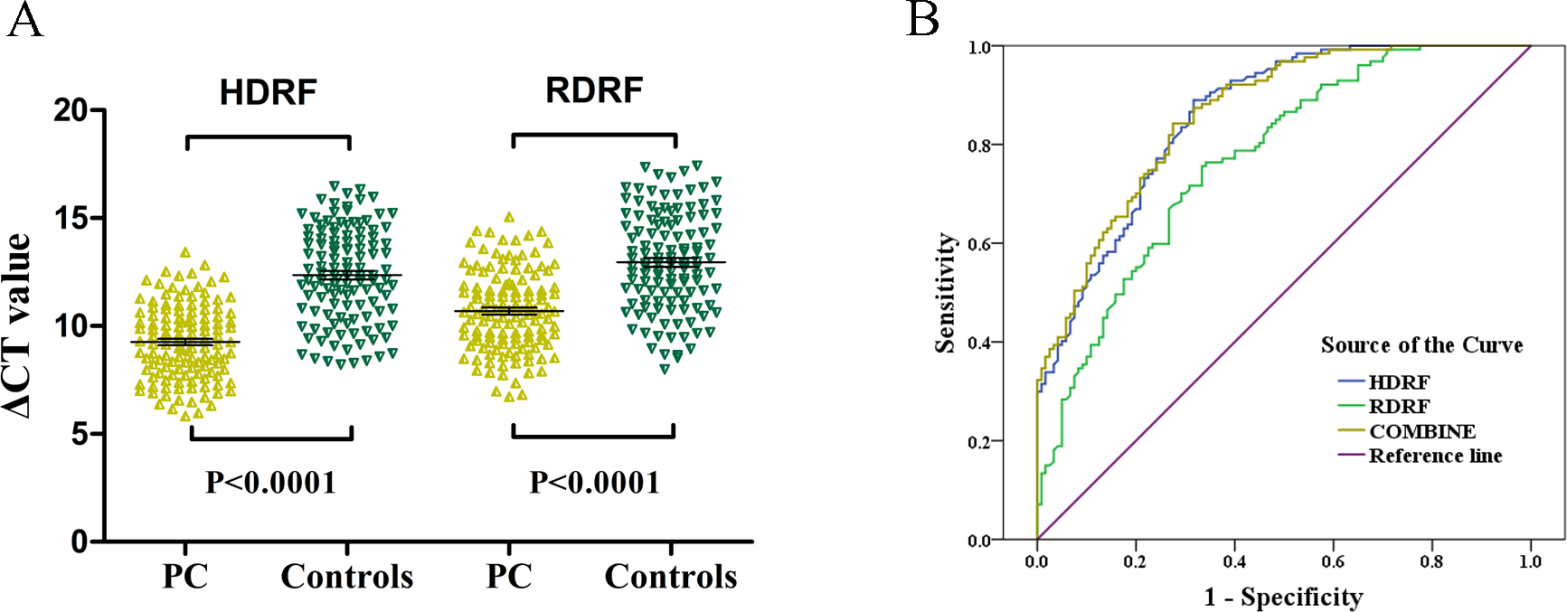
Validation of HDRF and RDRF as plasma-based biomarkers for detecting PC **A.** Scatter plot showing the HDRF and RDRF ΔCT values as measured by qRT-PCR in 127 PC and 122 control plasma samples. Smaller ΔCT value indicates higher expression. GAPDH was used as the normalization control. **B.** ROC curves showing the diagnostic performance of HDRF and RDRF.

To explore the potential role of HDRF and RDRF as plasma-based biomarkers for diagnosing PC, the HDRF and RDRF expression levels in plasma samples from 127 patients with PC and 122 healthy controls were measured using qRT-PCR. Compared to the controls, the HDRF and RDRF expression levels were significantly elevated in the patients (*p* < 0.0001) (Figure 8A). And the HDRF and RDRF levels were increased by an average of 9.59 and 6.78 fold-changes. Next, we constructed a receiver operating characteristic (ROC) curve by grouping all PC and normal samples into one class to examine the diagnostic performance of HDRF and RDRF. The areas under the ROC curves (AUC) were 0.857, 0.770, and 0.862 for HDRF, RDRF, and the combination of HDRF and RDRF, respectively (Figure 8B). The combined use of HDRF and RDRF slightly increased the diagnostic value. Further analysis of the diagnostic performance of HDRF and RDRF revealed that, at a cut-off of 11.4 and 11.925 (comparative threshold cycle [ΔCT] value), respectively, the sensitivity was 89% and 75.6% and specificity was 68.3% and 66.7%, respectively, for discriminating PC from non-PC. Our results indicate that HDRF and RDRF levels are promising indicators for distinguishing patients with PC from people without PC.

## DISCUSSION

### LncRNA expression profile of PC and its clinical significance

Several studies on lncRNA expression disorders in many cancer types have suggested that their abnormal expression might be a major cause of oncogenesis and that such cancers can be distinguished according to their altered lncRNA expression signatures [30, 31]. Microarray assay presents the possibility of efficient discovery of aberrantly expressed lncRNAs in different cancers. Recent reports have demonstrated that lncRNAs participate in PC development and progression by promoting cell growth, migration, invasion, and epithelial–mesenchymal transition [23, 25], indicating that they may play a critical role in the tumorigenesis of PC.

In this study, we describe an lncRNA expression profile associated with PC. We found that, among the significantly differentially expressed lncRNAs, HOTTIP-005, XLOC_006390, and RP11-567G11.1 were the most upregulated. A few studies have discovered the relationship between aberrant HOTTIP expression and hepatocellular carcinoma [16, 32, 33]. Recently, our research team and Cheng Y et al have reported that HOTTIP shows oncogenic-like activity in PC by enhances pancreatic cancer cell proliferation, survival and migration. But these two studies differ in the role of regulating HOXA13 [27, 34]. Nevertheless, XLOC_006390 and RP11-567G11.1 have not been identified in any cancers.

Intensive efforts have been expended to identify molecular predictive factors for survival in patients with cancer [35–37]. Our data show that HOTTIP-005, XLOC_006390, and RP11-567G11.1 are related to different clinicopathological features of PC and that HOTTIP-005 and RP11-567G11.1 overexpression are significantly correlated with lymph node metastasis and overall survival. Lymph node metastasis is an adverse pathological feature for overall survival in PC [38, 39]. Our results present the possibility that expression level of HOTTIP-005 and RP11-567G11.1 in tissues may serve as molecular prognostic biomarkers for selecting high-risk patients for further intensive treatment. Since the high lethality of PC, if we could find a biomarker that can potentially help in the early detection of this disease, the clinical significance will be greater. Therefore, we detected the level of HOTTIP-005 and RP11-567G11.1 fragments in plasma and the result will be discussed later.

We also found that XLOC_006390 overexpression was significantly correlated with neural invasion, a histopathological characteristic and an important prognostic factor of PC [40]. However, the mechanisms contributing to the perineural invasion and spread of cancer cells along the nerves remain poorly understood. This finding may indicate an approach for further research.

At present, serum CA19-9 is the only validated tumor marker for PC in widespread clinical use. But our statistical analysis showed that the overexpression of HOTTIP-005, XLOC_006390 and RP11-567G11.1 are all not correlated with it. For this result, we detailed analysis of the following reasons: First, CA19-9 and these lncRNAs are derived from different pathways, and they do not share the same mechanism of synthesis and secretion [41]. Second, the patients we enrolled were all at early stages that could undergo surgery, so these patients were not representative of patients with PC at all kind of stages. Furthermore, of patients with PC at early stages, the proportion that with elevated CA19-9 serum levels is very small [42, 43]. In order to research the relationship between CA19-9 levels and expression of these lncRNAs, maybe we should enroll patients with PC at different stages and perform Spearman's rank correlation.

### HOTTIP splice variant expression patterns in PC

Most lncRNAs have splice variants, but there are few studies on the expression pattern of these splice variants in different cancers. Consequently, the specificity and function of these expression patterns remain unknown. Shahryari et al. demonstrated that, in esophageal squamous cell carcinoma, two novel splice variants of SOX2OT: SOX2OT-S1 and SOX2OT-S2, are co-upregulated with *SOX2* and *OCT4*, and suggested that SOX2OT splice variants partly participate in tumor initiation and/or progression [44].

The expression pattern of HOTTIP splice variants has never been detected in cancer tissue. Ours is the first attempt to identify them in PC, and we found that HOTTIP-005 and HOTTIP-001 are the first and second most upregulated splice variants in PC, respectively. However, whether the expression pattern is PC-specific remains unclear, as does its potential involvement in PC initiation and progression, and further work is needed to elucidate these areas.

### The value of HOTTIP-005 and RP11-567G11.1 as plasma-based biomarkers for PC detection

Identifying proper biomarkers is a major aim in cancer research. The ideal biomarker should have the key characteristics of early detection, be minimally invasive, and have high specificity, sufficient sensitivity, and robustness. Several studies have investigated microRNAs in body fluids as potential biomarkers for the diagnosis or recurrence of PC. However, there is a lack of consistent results between these studies [45–47]. Recently, many studies have addressed the possible use of lncRNAs as biomarkers. However, most of them used tissue samples. And the diagnostic utility of circulating lncRNAs in PC has never been explored. As the lncRNA sequence length is very long, it is unlikely that lncRNAs exist in primary form in body fluids. Hence, we postulate that plasma lncRNAs probably exist in fragment form. Thereafter, our results, which are consistent with that of Ren et al. [20], validated this assumption.

To perform normal biological function, HOTTIP-005 and RP11-567G11.1 expression can be detected in normal tissues or cells, so it should be no surprise that the lncRNA-derived fragments HDRF and RDRF can also be detected in plasma of healthy subjects. However, the high levels of HDRF and RDRF were confirmed to derive from PC. In addition, we confirmed that HDRF and RDRF are stable. These features all present the possibility that HDRF and RDRF are diagnostic biomarkers of PC. Thus, the ROC analysis showed that as biomarkers, HDRF and RDRF both have relatively high sensitivity and specificity, especially HDRF. As the patients we enrolled were all at early stage of PC, it may explain the sensitivity and specificity of HDRF and RDRF was not so sufficiently high. To date, there is no specific biomarker for diagnosing PC, let alone early diagnosis; CA 19-9 is used as biomarker in the clinic generally. However CA 19-9 level testing has no utility as a screening tool in asymptomatic patients. Even among patients with symptoms suspicious for pancreatic cancer, elevated CA 19-9 is a poor predictor of pancreatic cancer with a predictive value of 0.5–0.9% [42]. This study aimed to improve this situation, detecting HDRF and RDRF or combining them with CA 19-9 may increase the efficiency of PC diagnosis. However, large-scale prospective studies are needed to further validate our findings.

## MATERIALS AND METHODS

### Ethics statement

Investigation has been conducted in accordance with the ethical standards and according to the Declaration of Helsinki and according to national and international guidelines and has been approved by the authors’ institutional review board. Prior to sample collection, we obtained informed consent from the patients and the healthy controls, and approval from the institutional ethics committees of Sun Yat-Sen Memorial Hospital.

### Patients and sample collection

144 cases of PC specimens (tumor and paired adjacent non-tumorous tissues) and four cases of CP specimens were obtained from patients undergoing surgical resection at the Sun Yat-Sen Memorial Hospital of Sun Yat-sen University from July 2006 to June 2014. All samples were immediately preserved in liquid nitrogen after removal. The histopathological diagnoses of all patients were clear and definite. No patient had received any form of anti-cancer treatment before surgery. The clinical follow-up time ranged 3–60 months. Overall survival was defined as the interval from the date of diagnosis to PC-related death. The clinical characteristics of all patients are listed in Table 2.

Whole blood samples were collected from the abovementioned patients and from 130 healthy controls at the Sun Yat-Sen Memorial Hospital examination center. For serum collection, the blood samples were allowed to coagulate for about 30 min to 2 h at room temperature. EDTA was used as an anticoagulant for the plasma collection, and samples were processed within 1 h of blood collection. All samples were centrifuged at 2000 × *g* for 10 min at 4°C in a refrigerated centrifuge. The supernatant (plasma/serum) was transferred to a fresh tube, leaving behind a 0.5-cm layer of supernatant to avoid disturbing the pellet.

### RNA isolation

Total RNA was extracted from fresh cultured PC cell lines or tissue samples using TRIzol (Invitrogen, Carlsbad, CA) according to the manufacturer's instructions. Serum/plasma RNA extraction was performed using TRIzol LS (Invitrogen). Briefly, 300 μl serum/plasma was mixed with 900 μl TRIzol LS. After vortex-mixing for 30 sec and standing for 5 min, 200 μl chloroform was added. The TRIzol–chloroform mixture was vortex-mixed for 15 sec, and the supernatant was discarded after centrifuging at 12,000 × *g* for 15 min at 4°C. The mixture was centrifuged again following the addition of absolute ethyl alcohol to clean the precipitate. Lastly, 10 μl diethylpyrocarbonate-treated water was added to the dry precipitate to entirely dissolve the total RNA. The total RNA extracted from 1 ml plasma/serum is approximately < 500 ng. Samples yielding > 500 ng total RNA were excluded to avoid genomic DNA contamination. Of the plasma/serum from the 144 patients and 130 controls, the total RNA of an eventual 127 patients and 122 controls were used for the study.

### LncRNA microarray analysis

We conducted lncRNA microarray analysis which was mentioned previous [27] on eight PC tissues(including four cases of diabetes mellitus–associated PC) and four chronic pancreatitis tissues utilizing a microarray targeting 7419 lncRNAs (Arraystar Human LncRNA Microarray V3; Agilent Technology, Santa Clara, CA). The Agilent microarray analysis protocol was used for sample labeling and array hybridization with minor modification. The raw data were analyzed utilizing Agilent Feature Extraction software. The robust multiarray average algorithm was used to adjust the background signals. Normalized data were obtained using the quantile method of intra-microarray normalization and the median method of baseline inter-microarray transformation between the microarrays. Differentially expressed genes with a raw expression level of > 400 in more than 4 out of the 12 samples used for profiling were extracted and ordered by *p*-value. The microarray platform and data were submitted to the Gene Expression Omnibus (GEO) public database at the National Center for Biotechnology Information (accession number: GSE61166, http://www.ncbi.nlm.nih.gov/geo/query/acc.cgi?acc=GSE61166).

### qRT-PCR

Total RNA was converted to complementary DNA by reverse-transcription using oligodT primers and SuperScript II reverse transcriptase (Invitrogen). For qRT-PCR, three replicates per sample were amplified and analyzed using a Roche Light-Cycler (Basel, Switzerland). Reactions were carried out in a 20-μl volume using SYBR Green Reaction Mix (Qiagen Science, GER) with 0.5 mM primer. To detect the lncRNA expression levels in the tissues and PC cell lines, β-actin was used as the normalization control. There are no established endogenous plasma mRNA control values for normalizing plasma or serum lncRNA fragments. Therefore, to quantify plasma/serum lncRNA fragments, we first measured glyceraldehyde-3-phosphate dehydrogenase (GAPDH) and β-actin expression in the plasma/serum from 30 patients with PC and 30 healthy controls: GAPDH and β-actin were stably expressed in all plasma/serum samples (Supplementary Figure S4). However, plasma/serum GAPDH expression was higher than that of β-actin. Consequently, GAPDH was identified as a stable reference and used to normalize the plasma/serum lncRNA fragments. The relative gene expression levels were determined using the comparative threshold cycle (2 − ΔΔCT) method. All results are expressed as the means ± standard deviation of each independent experiment. The gene-specific sequence information of the qRT-PCR primers are listed in Supplementary Table S1.

### Cell culture

The human PC cell lines PANC-1, BxPC-3, Capan2, MIAPaCa-2, SW1990, and AsPC-1 were purchased from American Type Culture Collection (Manassas, VA) and grown in complete growth medium, as recommended by the manufacturer, and with 10% fetal bovine serum and 1% penicillin/streptomycin. HPDE6 cells (immortalized human pancreatic ductal epithelial cells) were obtained from Dr. SN Zhang (Sun Yat-Sen University, Guangdong, China). All cells were cultured in a humidified 5% CO_2_ incubator at 37°C.

### Xenograft experiments

All experiments involving animals were conducted according to the institutional guidelines of Guangdong Province and were approved by the Use Committee for Animal Care. SW1990 cells (3 × 10^6^, 200 μl) were injected subcutaneously into the dorsal flank of BALB/c nude mice (4–6 weeks old). The control mice received mock injections of 200 μl 50% basement membrane matrix in Hank's balanced salt solution. Each of the two groups contained 10 mice. After four weeks, the mice were sacrificed and their blood was collected in EDTA-anticoagulant tubes by cardiac puncture.

### Statistical analysis

The comparison of lncRNA expression levels between the groups was analyzed using Student's *t* test. The correlation between lncRNA and clinicopathological characteristics was analyzed using the Pearson chi-square test. Overall survival was assessed using the Kaplan–Meier method; differences in survival rates were assessed with log-rank testing. A ROC curve was established to evaluate the diagnostic value of HDRF and RDRF. The AUC was used to assess the predictive power. All tests were two-sided, and *p* < 0.05 was considered statistically significant. All statistical analyses were performed using SPSS 18.0 and GraphPad Prism 5 software.

## CONCLUSIONS

Our results suggest that HOTTIP-005 and RP11-567G11.1 are associated with the prognosis of PC, that HOTTIP-005 and RP11-567G11.1 fragments can be detected in human plasma in remarkably stable form, and that these fragments may have the potential to be used as biomarkers to distinguish PC from non-PC. Nevertheless, further large-scale and prospective studies are needed to confirm our findings.

## SUPPLEMENTARY FIGURES AND TABLES


