# Supplementary material for: Expression profile of long non-coding RNAs in pancreatic cancer and their clinical significance as biomarkers
Source: Oncotarget. 2015 Oct 2;6(34):35684–98. doi: 10.18632/oncotarget.5533 (PMC4742134; doi:10.18632/oncotarget.5533)
Supplement: Supplementary file 1 [file oncotarget-06-35684-s001.pdf]

SUPPLEMENTARY FIGURES AND TABLES

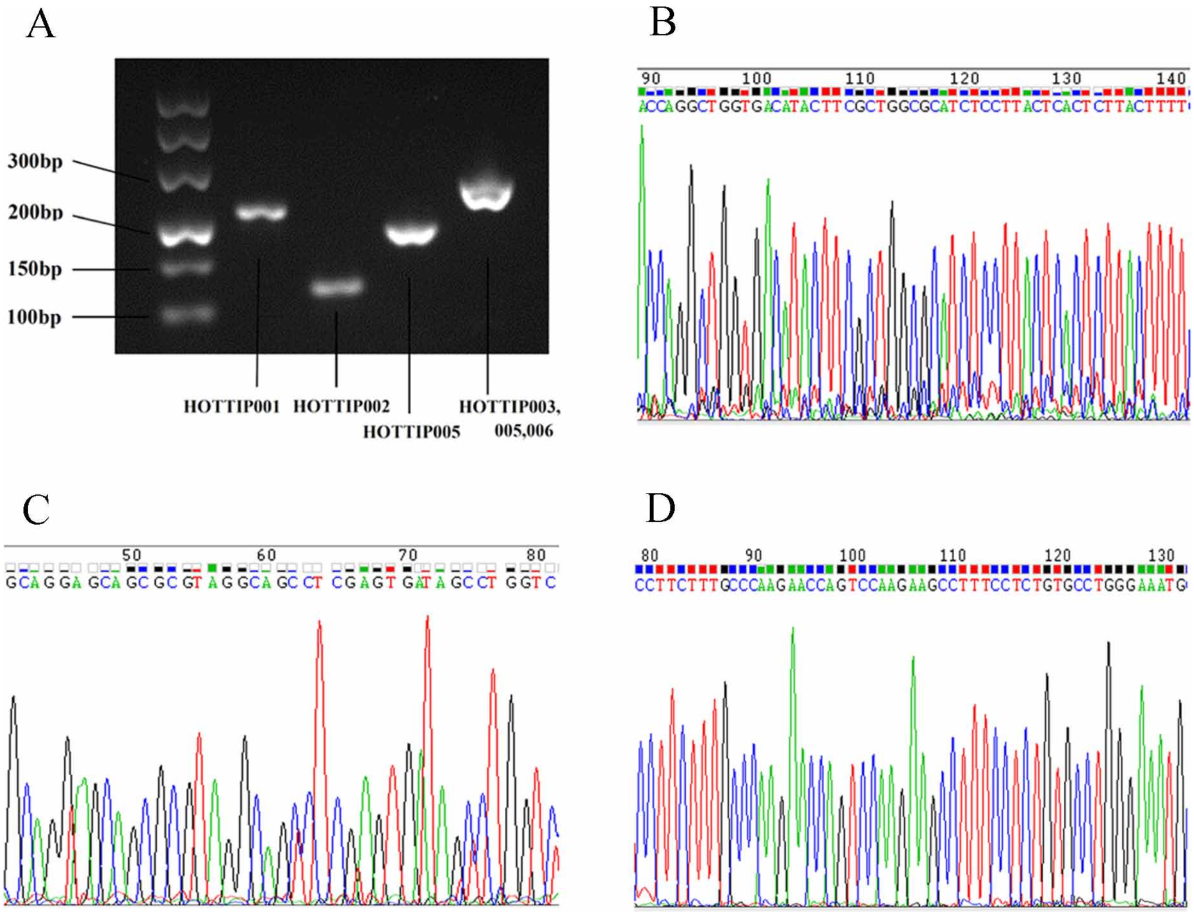

Supplementary Figure S1: Validation of HOTTIP-001, HOTTIP-002, and HOTTIP-005 qRT-PCR product specificity. A. Agarose gel electrophoresis of HOTTIP-001, HOTTIP-002, HOTTIP-005, and HOTTIP-003,005,006 qRT-PCR products. **B–D.** Sequencing results of HOTTIP-001, HOTTIP-002, and HOTTIP-005 qRT-PCR products.

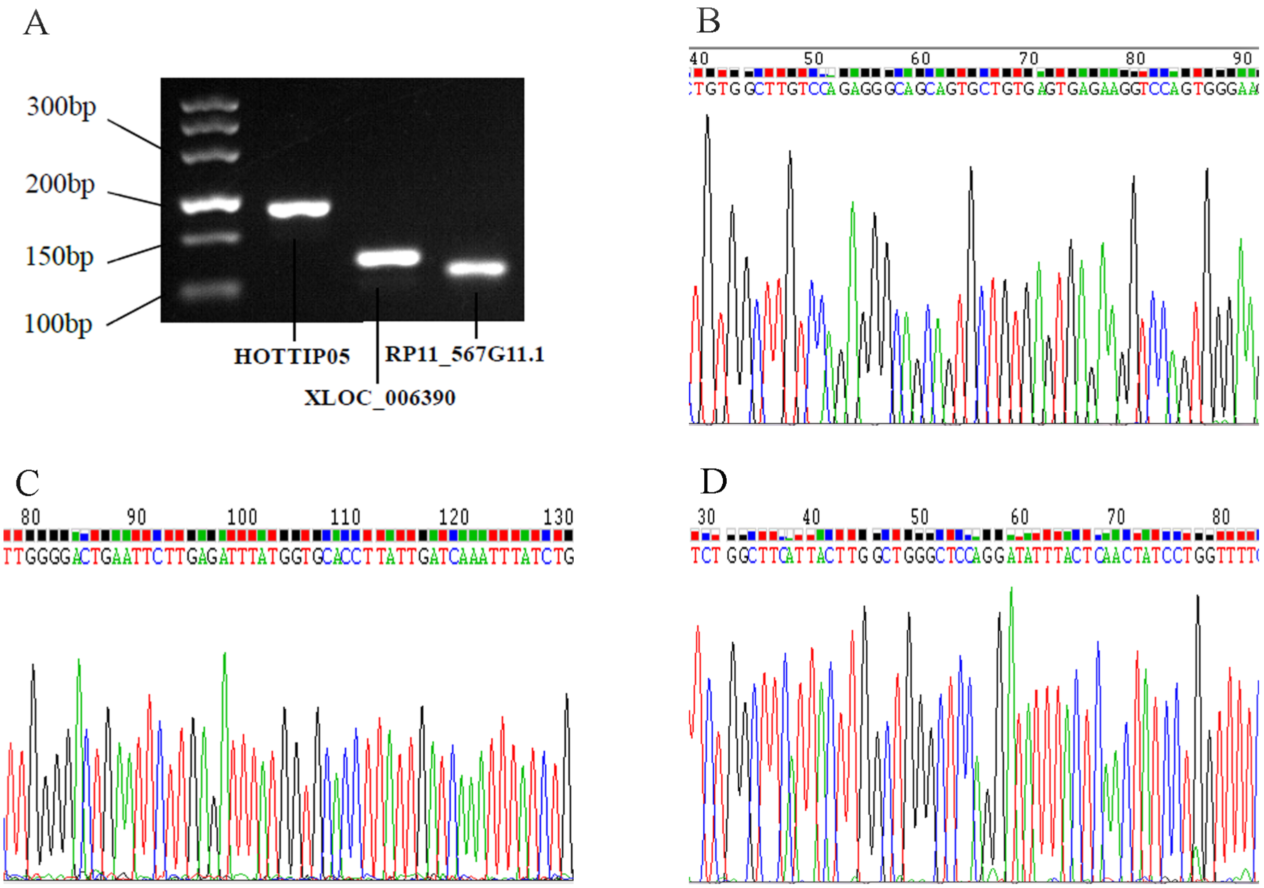

Supplementary Figure S2: Validation of XLOC_006390, HOTTIP-005, and RP11-567G11.1 qRT-PCR product specificity. A. Agarose gel electrophoresis of XLOC_006390, HOTTIP-005, and RP11-567G11.1 qRT-PCR products. **B.** XLOC_006390, **C.** HOTTIP-005, and **D.** RP11-567G11.1 qRT-PCR product sequencing results.

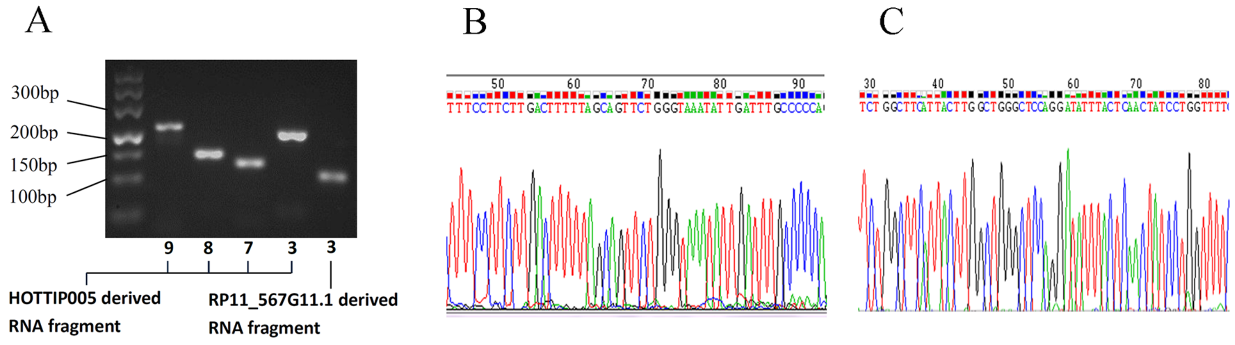

Supplementary Figure S3: Validation of HDRF and RDRF qRT-PCR product specificity. A. Agarose gel electrophoresis of HOTTIP-005 and RP11-567G11.1 derived fragments qRT-PCR products. **B.** Sequencing results of HDRF qRT-PCR product. **C.** Sequencing results of RDRF qRT-PCR product.

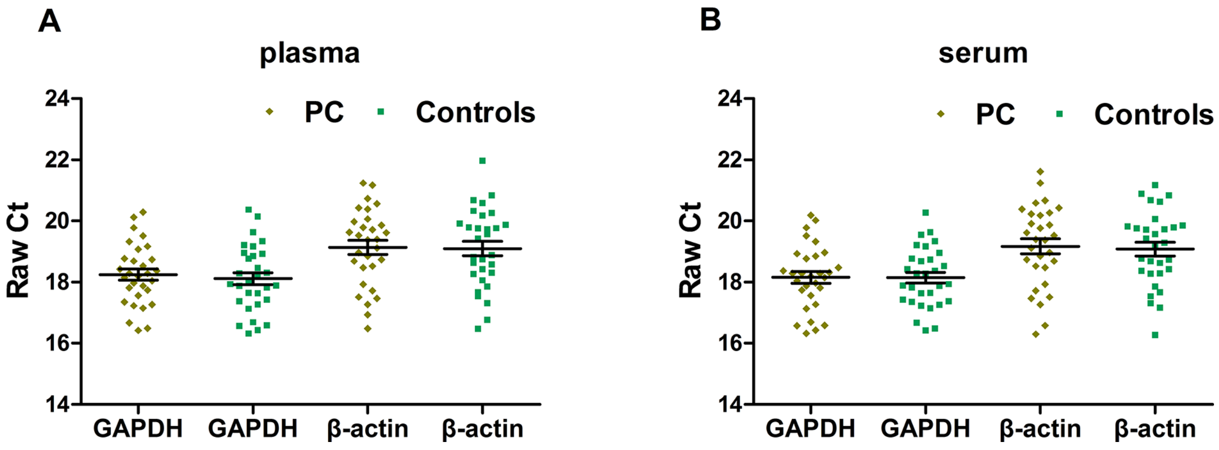

Supplementary Figure S4: Raw CT values of plasma and serum GAPDH and β-actin. Expression of the candidate reference genes *GAPDH* and β-actin (*ACTB*) were measured in the plasma **A.** and serum **B.** from patients with PC (n = 30) and healthy controls (n = 30). Kruskal-Wallis testing revealed no significant difference between the two groups. Plasma/serum *GAPDH* expression levels were higher than that of *ACTB*.

Supplementary Table S1: Primers used in qRT-PCR

Primer name		Sequence (5′—3′)	Product length (bp)
β-actin	sense	CATGTACGTTGCTATCCAGGC	236
	antisense	CTCCTTAATGTCACGCACGAT	
GAPDH	sense	CGCTGAGTACGTCGTGGAGTC	172
	antisense	GCTGATGATCTTGAGGCTGTTGTC	
HOTTIP-005	sense	CACAAGCAGGTTTGTCTGAGAGG	187
	antisense	TAGGGACACATCGGGGAACTA	
XLOC_006390	sense	TCCTTTGAATCCCTGAGAACTGAAC	126
	antisense	ACCTTCCTTCCCACTGGACCTTC	
RP11_567G11.1	sense	TGGAAAACCAGGATAGTTGAGT	117
	antisense	ATCCCTTACAGACCCTTTATTG	
HOTTIP-001	sense	ACGCATATTCACGCATCA	209
	antisense	TTACCAAGCCACAGGAGA	
HOTTIP-002	sense	GGAAGGTAGTTAGTTCTGAAC	115
	antisense	TTGGACCAGGCTATCACT	
HOTTIP005-1	sense	TTGGGTTGCGATCTGGAGCAG	102
	antisense	CAACTCCATCGAAGCCGAAG	
HOTTIP005-2	sense	GAGACCACAGGACGGACATCG	182
	antisense	CAGTGTGGACAGGGAAGGGATAG	
HOTTIP005-3	sense	TGTCCACACTGTTAGTGAGCAAT	194
	antisense	ACAGGGATCGCTCGCTCTATCTC	
HOTTIP005-4	sense	TTGAGATAGAGCGAGCGATCC	202
	antisense	AATAGCGGGTTCCCAGATTCC	
HOTTIP005-5	sense	GGGAACCCGCTATTTCACTCTATT	252
	antisense	AGAACCCCTCGACAAAACATG	
HOTTIP005-6	sense	CAAAGTGAAAGTGGGCACATTACC	187
	antisense	GCCTTAGACAGCAGATGGTGG	
HOTTIP005-7	sense	ACCAATGTAAGTGTCGCCCAATA	124
	antisense	AAACTGGGGGCAAATCAATAT	
HOTTIP005-8	sense	TTCCTCCCTCCAAGTGGCATT	143
	antisense	ACTGCAACTTTCAACTTGACCTTG	
HOTTIP005-9	sense	TCTGCCTCCGCCCTGCTCA	209
	antisense	AGGGTTCGGGGCTACGGTGAA	
HOTTIP005-10	sense	CACAAGCAGGTTTGTCTGAGAGG	187
	antisense	TAGGGACACATCGGGGAACTA	
RP11_567G11.1-1	sense	ATCTACTGATTGGATGAGGCC	95

(*Continued*)

Primer name		Sequence (5′—3′)	Product length (bp)
	antisense	CTGTGTGGGATAACTTTAGGCA	
RP11_567G11.1-2	sense	CCACCAGCATTCTGAAAGACAAG	124
	antisense	TGGGCTCCAGGATATTTACTCAA	
RP11_567G11.1-3	sense	TGGAAAACCAGGATAGTTGAGT	117
	antisense	ATCCCTTACAGACCCTTTATTG	
